# The Role of Distributed Health Literacy in Asthma Integrated Care: A Public Medical Context from Portugal

**DOI:** 10.5334/ijic.3301

**Published:** 2018-06-05

**Authors:** Liliana Abreu, João Arriscado Nunes, Peter Taylor, Susana Silva

**Affiliations:** 1 ISPUP-EPI Unit, Instituto de Saúde Pública, Universidade do Porto, Rua das Taipas, n˚ 135, 4050-600 Porto, PT; 2 i3S – Instituto de Investigação e Inovação em Saúde, Universidade do Porto, Rua Alfredo Allen, Porto, PT; 3 Faculdade de Medicina, Universidade do Porto, Porto, PT; 4 Center for Social Studies and School of Economics of the University of Coimbra, PT; 5 Science, Technology and Values Program, University of Massachusetts, Boston, US

**Keywords:** health mediators, health literacy, people-centred care, integrated care, asthma

## Abstract

**Background:**

Improvements in asthma integrated care might be achieved through in-depth knowledge about how health literacy is dispersed through a group. This study intends to map out health literacy mediators (those who *makes his/her literacy skills available to others for them to accomplish specific literacy purposes)* and how they enable self-management skills in patients with asthma.

**Methods:**

Twenty interviews were conducted in a Primary Care Center of Porto using the McGill Illness Narrative Interview. Data were thematically analyzed as case-based and process-tracing-oriented.

**Results:**

Interviewees with a dense network of mediators revealed a low impact of asthma on their lives, dependence on primary care physician for instrumental support and dependence on family members to provide emotional/pragmatic support. Interviewees who relied on a restricted network of mediators (belonging to formal sources of health services and providing informational support) described episodes of crisis as disruptive and demonstrated a reactive approach to self-management skills.

**Conclusions:**

The roles performed by core health mediators (health professionals, family/friends, media) in support of asthma management varied according to patients’ narratives of minimization/disruption, connected to dense/restricted social networks. To clarify the boundaries of responsibility-shifting and to enrich support provided by formal sources of health services and peer education groups is needed.

## Introduction

Therapeutics and asthma patient-centred care have advanced over the last two decades [1,2,3,4]. However, asthma is still often poorly managed [5,6], with asthmatics having difficulties in access or use of health care and in adhering to treatment regimens [5], as well as lacking self-management skills [7,8]. Asthma is still an important reason for hospital admission worldwide [9,10] and causes considerable limitations on the lives of both patients and their families [11,12]. These barriers pose significant direct and indirect economic costs and psychosocial burden [13], particularly evident in asthmatics with inadequate health literacy [14]. Instrumental and emotional support provided by robust social networks and informal caregivers is likely to overcome such barriers [15,16] by assisting asthmatics in obtaining, processing, understanding and applying basic health information and services needed to make appropriate health decisions and meet daily self-management demands [17,18,19]. Improvements in asthma integrated care might thus be achieved through in-depth knowledge about how health literacy is dispersed through a group of individuals or a community is considered – distributed health literacy – as coined by Edwards et al. [20]. It is particular critical for health literacy interventions in communities to identify health mediators and their roles in an asthmatic’s tasks related to health literacy, that is, anyone or anything which ‘*makes his, her or its literacy skills available to others, on a formal or informal basis, for them to accomplish specific literacy purposes*’ [21], such as the use of regular inhaled preventive or prophylactic medication namely, help to quit smoking, motivation to practice physical exercise and lost weight, and help in avoiding professionals risk environments [22].

Pioneering case studies in India highlighted the influence of communities on health practices, showing for instance that sharing information and experiences among mothers living in the same village increased the probability of immunizing their children, with this factor being stronger correlated with childhood immunization rates rather than the individual literacy level of mothers [23,24]. In fact, daily life occurs in circumstances where health-related decisions and behaviors are not made just by individuals but are powerfully influenced by family members, peers and/or community leaders [25,26].

Among patients living a long-term health condition, such as asthma, distributed health literacy emerges as a potential resource for managing one’s health, communicating with health professionals and making health decisions [20]. This approach epitomizes the current worldwide movement towards integrated, people-centred health services [27], co-production of knowledge, shared care and shared governance for health sustaining the provision of tailored health services that aim to synchronize care both with and around the needs of service users, their families and the communities, meeting an individual’s or group’s specific characteristics within the context of their lives [27]. To map individuals and resources involved in patients’ asthma management and exploring the properties of relations and ties between health mediators regarding density, range, boundedness, and homogeneity [28], it is essential to describe the exclusive forms, organization and composition of patients ‘health networks’ [27]. These networks are often important conduits for shared resources whose enhancement must also reflect the views of patients to certify that the healthcare services and health governance consider their beliefs, contexts and needs [29].

This study focuses on how asthmatics draw on their social network for support with health literacy-related tasks, mapping out health-literacy mediators for each individual, and how they enable self-management skills and knowledge about asthma.

## Methods

This is a qualitative observational study conducted in Portugal, where the first National Asthma Control Program was established in 2000, aiming at improving skills and competences in the patients and their families [30]. In 2010, the only Portuguese National Asthma Survey – Inquérito Nacional sobre Asma (INAsma) estimated a prevalence of ‘current asthma’ (i.e. with symptoms in the last 12 months) in the Portuguese population of 6.8% (95% Confidence Interval 6.0–7.7); among these, 57% had the disease under control [31].

In Portugal, asthma access care is made through standard primary care, in medical appointments without standardized routines for asthma care (**Box 1**). Under the presence of a restrict number of clinical criteria, patients are referred to specialized allergy consultations in the hospitals. Almost half of the 225 hospitals located in Portugal are private (n = 111), being frequently used by people with higher socioeconomic status (SES) to treat asthma.

**Box 1: Brief description of the Family Health Unit (USF) in the study**

**Table:** 

**History and nature in the health system**	Reform of primary health care in Portugal 2005 – Constitution of USFs, coordinated by one of the five Health Center Clusters (ACeS). ‘Bottom-up’ approach – self-organized teams of health professionals through voluntary applications to provide care in a particular geographic region (Ministry of Health, 2010). Each team has autonomy in terms of technique-care and functional management and it is constituted by family doctors, nurses and clinical secretaries. There are 483 USFs in Portugal, with 8945 professionals (*data from 5th May 2017*). Each doctor working on the USF of this case study provide primary care to 1800 patients (legal max. 1900). Public primary health care units are the first contact point of the citizen with the health system.
**Provision of asthma care – guidelines**	**Content of asthma care:** Total control of symptoms; reduction of risks of crisis; reduction of progressive bronchial obstruction and adverse effects of medication; diagnosis and control of comorbidities; promote therapeutic adherence; correct use of inhalers; improvement of quality of life; and support for having a daily life without limitations. **Role of family doctor:** Guarantee that all asthmatics get an adequate and personalized healthcare; raise awareness and provide essential information about the disease to all citizens; promote therapeutic adherence and reinforce the importance of asthmatic empowerment and family support in asthma management; longitudinal relationship with patients, continuous care; reevaluate an asthma without control and refer these cases to a specialist in pulmonology and immunoallergology.

This study was carried out in a Family Health Unit located in an urban area in Porto District (Northern Portugal). From October 2014 to December 2015, a convenience sample of adults diagnosed with asthma, for more than one year, were invited by all health professionals working at this center (six primary care physicians and five nurses) to participate in the study after medical consultations (not necessarily related to asthma). In this center, 337 out of 10 361 users (including adults and children) had been diagnosed with asthma (Data from 2015, provided by the Primary Care Center). Invitations were made during the days the researcher went to the center. Participants were purposively sampled to include patients with a diagnosis of asthma in childhood and in adulthood, under the assumption that early age asthma onset might distinguish different asthma phenotypes [32], which may be related to persistence and severity of disease and directly linked to different self-management experiences. Heterogeneity sampling was used for maximum variation of views and experiences regarding adherence to asthma self-management, until the point when no new, significant data emerged from data analysis – that is, the point of theoretical saturation [33], possible to achieve with at least 15 interviews in nonprobabilistic samples [34]. All the 20 invitees accepted to participate, and 4 asked to reschedule the interview for another day of their convenience.

Data were collected based on the McGill Illness Narrative Interview (MINI), previously applied in a Portuguese study about health knowledge of people with asthma and breast cancer [35]. MINI is a semi-structured ethnographic interview schedule, intended to produce narratives and status of health knowledge [36], comprising the next sections: 1) Initial Narrative – purposefully unstructured, letting interviewees to tell their story freely; 2) Prototypes – structured, intending to elicit narratives on typical experience of self and others; 3) Explanatory models – causal type of reasoning; 4) Help seeking and service utilization – interviewee experience with health services and treatments; 5) Impact of illness – explore if and how patients believe the illness has led to changes in their identity and way of life since its diagnosis. For the purposes of this paper, two extra topic questions were addressed to explore the role of health mediators: “Do you usually go accompanied to the medical visits (if yes, with whom)? If we ask you to choose someone to help you in a health-related issue, who would you choose and why?” (it was stated to the patients that they could mention more than one person).

Interviews were conducted by the first author in a reserved room at the Primary Care Center, and lasted 50 minutes on average. All were taped, professionally transcribed verbatim, and checked for accuracy.

Data were thematically analyzed [37] as case-based and process-tracing-oriented, by the first author, with the assistance of NVivo 10 (QSR International, USA, 2013). Process-tracing was accomplished through coding each interview to identify categories associated with the following previously defined themes of interest [38], incorporating constant comparison of the coded interviews, and exploration of deviant cases: 1) ‘dealing with an asthma diagnosis’, which explored attitudes, trajectories and levels of awareness and knowledge about asthma; 2) ‘self-management skills’, when discourses pointed to access to health services and how participants manage medication, and potential triggers of asthma crisis; and 3) ‘health literacy mediators’, where the interviewees mentioned sources of support (formal, informal or other) and types of support (pragmatic, informational and emotional). The development of the coding framework and selective coding was discussed by three of the authors. Disagreement was solved by continuous and iterative joint discussion until consensus could be reached.

The 20 patients selected consisted of 17 women (12 elementary educational level, 3 high education level and 2 secondary educational level) and 3 men (2 elementary educational level and 1 secondary educational level). Their ages ranged from 21 to 70 years old. The diagnosis at adulthood length ranged from one to 20 years and five were diagnosed in childhood (see Table 1).

**Table 1:** Interviewees’ characteristics, according to awareness narrative.

Pseudonym	Age	Educational level	Household members	Diagnosis	Asthma in the family

**Narrative of minimization**					

Ana	61	Elementary	Husband	Adulthood (at 60)	Yes (son and brother)
Graça	45	Elementary	Husband	Adulthood (at 39)	Yes (mother, husband)
Maria	58	Elementary	Husband	Adulthood (at 46)	No
Cristina	34	High	Husband and son	Adulthood (at 27)	Yes (sister)
Filipa	59	High	Husband	Adulthood (at 56)	Yes (husband)
Sebastião	54	Elementary	Wife	Adulthood (at 51)	Yes (mother, daughter)
João	45	Elementary	Wife	Adulthood (at 42)	No
Elsa	46	Secondary	Husband and son	Adulthood (at 41)	Yes (father and son)
Joana	53	Elementary	Husband	Adulthood (at 39)	No
Júlia	21	Secondary	Husband and son	Adulthood (at 19)	Yes (father)
Manuela	65	Elementary	Son	Adulthood (at 61)	Yes (mother and sister)
Ema	62	Elementary	Husband	Adulthood (at 57)	No

**Narrative of disruption**					

Laura	68	Elementary	Mother and husband	Adulthood (at 58)	No
Olinda	65	Elementary	Alone	Childhood	Yes (grandmother)
Rita	52	Elementary	Husband	Adulthood (at 32)	Yes (grandmother)
Helena	66	Elementary	Husband	Childhood	Yes (aunt)
Anabela	31	High	Husband and son	Childhood	Yes (sister recently diagnosed)
António	22	Secondary	Parents	Childhood	Yes (sister)
Idalina	70	Elementary	Alone	Adulthood (at 65)	Yes (grandmother)
Isabel	30	Secondary	Boyfriend	Childhood	Yes (son)


All participants formalized their collaboration through a written informed consent. Ethical approval was granted by the Research Ethics Committee of the Institute of Public Health of the University of Porto.

## Results

Two distinct narratives emerged from data analysis – the narrative of minimization and the narrative of disruption. The narrative of minimization was enacted by interviewees who had a dense network of health literacy mediators. These patients claimed low impact of asthma on their lives and daily routines, easy control of symptoms and avoidance of major crisis, and dependence on their primary care physician for instrumental support and on close family members with asthma to provide emotional and pragmatic support with medication and alert them to situations that might trigger an asthma attack. A narrative of disruption was enacted by interviewees who relied on a restricted network of core health mediators made up of formal sources of health services (clinical interaction or online) used mainly to provide informational support. They described episodes of crisis as highly disruptive, their difficulties in controlling crises and their feelings of stigma. These patients tended to hide asthma and to look for alternative and complementary solutions to control anxiety, demonstrating a reactive approach to asthma management. Results are illustrated by direct anonymized quotes, translated by the authors, drawn from the interviews. These are presented in Table 2 (narrative of minimization) and Table 3 (narrative of disruption).

**Table 2:** Representative quotes of the main themes – narrative of minimization.


**1.1 Dealing with an asthma diagnosis** (low impact; family condition; importance of the diagnostic consultation)	

[1.1a]	Elsa: “I was not surprised (when diagnosed with asthma) … My son has asthma since a child, my father also had it. It is in the family.”
[1.1b]	Júlia: “I didn’t worry (about asthma diagnosis). Because I was always feeling bad (…) and then I saw the problem solved.”
[1.1c]	Filipa: “I already knew that he [family doctor] was also asthmatic (…) and he said: “don’t be afraid, because when I was in college I already had asthma and I am still around.” To have heard this was reassuring.”
[1.1d]	Filipa: “I already knew that he [family doctor] was also asthmatic (…) he said to me: ‘don’t be afraid, because when I was in college I already had asthma and I am still around.’”

**1.2 Self-management skills** (avoidance of major crises; control of symptoms by SOS medication)	

[1.2a]	Elsa: “I know that if I am in some bad environment, with smells, of course I will be attacked. But I know what to do (SOS medication).”
[1.2b]	Júlia: “It is more at night that I have more asthma, (…) and when I am attacked, I take my SOS pump and I immediately get better.”
[1.2c]	Maria: “My doctor prescribed me a medication, and I started doing it. But then, my mother-in-law, who suffers from bronchitis and used to take the same medication, told me: ‘don’t take that, then you get used to it and can’t walk anymore’: So I stopped. But then I went to a pharmacy and he (pharmacist) told me to do it, and I did; but later another pharmacist, in another pharmacy, told me to leave it. And now I don’t take it.”

**1.3 Health literacy mediators** (dense network)	

1.3.1 Family and friends (close family members; emotional and pragmatic support)	

[1.3.1a]	Filipa: “The medication was the same as my husband, and sometimes we shared. (…) When we go on vacations, his last question before leaving home (…) is if I have brought the pumps.”
[1.3.1b]	Manuela: “I went there 42 because they (siblings) told me that he (a doctor) was great! (…) He did an exam that nobody here in Portugal told me to do.”
[1.3.1c]	Graça: “If I’m having a crisis, (…) my daughters (…) know exactly what to do: one of them goes right away search for my inhaler/pump. They know that I always carry one in my bag or in my pocket.”
[1.3.1d]	João: “If I am a little ‘attacked’ my wife immediately says: “you will have a crisis!”. (…) She always ensures that I take it (medication).”
[1.3.1e]	João: “Once, my friends and I went to ride in karts. The building, completely indoor, was full of smoke from the karts. I felt bad, completely short of breath (…) My friends came with me outside, to breathe.”
[1.3.1f]	Filipa: “I have friends calling me, saying: “So, did you go to that doctor?” (…) They worry.”

1.3.2 Health professionals (PCP; instrumental support)	

[1.3.2a]	Sebastião: “I go there (primary care center) often (…) usually I have two consultations a year.”
[1.3.2b]	Ema: “I don’t like to go to different doctors, because they all say different things.”
[1.3.2c]	Cristina: “My doctor is great. She really worries about us; we can feel it is genuine. She has a very close relationship with me, my son… I already told my sister [also asthmatic] she should move to this primary care center and be patient of my doctor.”

1.3.3 Media (not always reliable)	

[1.3.3a]	Cristina: “I also go to the internet (…), but I think that sometimes it is bad, because they give opinions, but they are not experts.”
[1.3.3b]	Graça: “If people say things differently from the doctors, it is wrong, I don’t trust it. Not everybody can write about this (asthma); it must be a doctor.”


**Table 3:** Representative quotes of the main themes – narrative of disruption.


**2.1 Dealing with an asthma diagnosis** (disruptive impact; feelings of stigma; to hide asthma)	

[2.1a]	Isabel: “Asthma did change my life and the way I see life… At least when I’m attacked.”
[2.1b]	António: “When practicing swing, soccer, running, and other sports, it (asthma) does not allow us to be as resistant as other persons.”
[2.1c]	Rita: “I want to do things, but I’m not able to do so (…) and people sometimes do not understand. Sometimes I feel people are saying: ‘she is faking it.’ (…) It is very upsetting.”
[2.1d]	Idalina: “It’s hard! People stay disgusted, with a weird face (when seeing an asthma attack).”
[2.1e]	Isabel: “He (participant’s son, also asthmatic) does not take it (the pump) to school (…) (because) he does not like to say he has asthma, since very little.”
[2.1f]	Laura: “Causes? I think it was from tobacco (her husband smokes). And I don’t smoke! Do you believe? It is so frustrating.”

**2.2 Self-management skills** (reactive approach; alternative solutions; feelings of personal guilt)	

[2.2a]	Rita: “I feel anxious and in panic [when having a crisis]. (…) Oh my God, if I use the pump and it does not work, I panic, and I just pray to pass.”
[2.2b]	Anabela: “Yes, I have searched for other options, such as, acupuncture. And it was good, I felt some benefits.”
[2.2c]	Helena: “I have the pump. My doctor told me to use it every day, but I only do it once in a while. Because I like to read patient information leaflet, and if everybody read that, people would not take medications. Because what it is written there, it really might happen.”
[2.2d]	Anabela: “I don’t know, maybe I’ve been lazy…The pediatrician of my son told me my medication (for asthma) was totally outdated and I should start a new treatment. I really have to convince myself and have a medical consultation about this.”
[2.2e]	Isabel: “People say that the beach is bad for the lungs, and when I’m attacked I don’t go to the beach. I’m afraid.”
[2.2f]	António: “The last couple of years, (…) I take medication more regularly (…) and I go to the emergency rooms quite often (…) It already happened go to the hospital twice a month.”
[2.2g]	Anabela: “Usually, when I’m attacked I take Ventilan in SOS, and it is effective. However, since last month I have been taking Ventilan 3 to 4 times a day, and it is not working, I still have the symptoms.”

**2.3 Health literacy mediators** (restricted network)	

2.3.1 Health professionals (PCP; communication issues)	

[2.3.1a]	António: “Sometimes I need to know something quite specific – for instance why my crisis are more regular – and I just ask to the doctor. But actually, nowadays, we don’t need to come here to know something, I go online or to the medical leaflets.”
[2.3.1b]	Rita: “I’m from the opinion that explaining things helps a lot. For instance, last month I went to do some exams at the hospital, and during the exam I asked a lot of things to the nurse. She was very nice, and answered me. But her colleague told me: ‘you don’t need to know! It’s the doctor that must know everything!’. But that’s wrong. We are the patients, we should know.”
[2.3.1c]	Ema: “I think the hardest part is the language, I think it is. Because doctors speak for each other’s, not for the patient. Once I was hospitalized, and a group of doctors just came to my room, they spoke for each other’s, and I didn’t understand a thing.”

2.3.2 Media (Internet; not reliable)	

[2.3.2a]	Anabela: “Once I was having cramps, so I went online to search something about what was causing me that, and read that people with asthma tend to have more cramps than usual. But I don’t know if it is true, I only find that in one website. No where else.”

2.3.3 Family and friends (lack of support)	

[2.3.3a]	Helena: “My husband usually tells me: ‘Calm down’! (…) When he tells me that, I got much worse. Oh my God, I can’t hear that. (…) I know that my husband just wants to help me, but I can’t avoid this.”
[2.3.3b]	Idalina: “Nobody helps or anything. I am the one who have to help the others.”


### Narrative of minimization

#### Dealing with an asthma diagnosis

Asthma was frequently mentioned as a condition that ‘is in the family’ and, consequently, a diagnosis of asthma was not a surprise [1.1a]. Most interviewees had close family members with asthma, whether parents, children, siblings or partners. Diagnosis emerged as a map for steps ahead and the validation of previous symptoms, and signaling the end of suffering now that treatment became possible [1.1b].

Interviewees suggested that the degree of familiarity with asthma determined how people cope with such a condition. By comparing asthma severity among relatives, interviewees tended to report feeling reassured about having a less severe asthma [1.1c]. The diagnostic consultation emerged as a landmark, with interviewees recalling the exact words of the primary care physician, mentioning the conversation as ‘reassuring’ [1.1d].

#### Self-management skills

Interviewees identified several self-management skills to avoid triggers of an asthma crisis and deal with crises. Patients recognized as triggers to asthma crises “bad environments”, such as places with intense smells or smoke [1.2a]. Although this group spoke of preventive behavior, the most prominent self-management skill described was to control the symptoms through quick relief medication [1.2a; 1.2b]. Participants described situations where they did not know if they should take a particular medication, in light of contradictory advices given by different people from their social networks – doctor, mother-in-law, and pharmacists [1.2c].

#### Health literacy mediators

**Figure 1:**
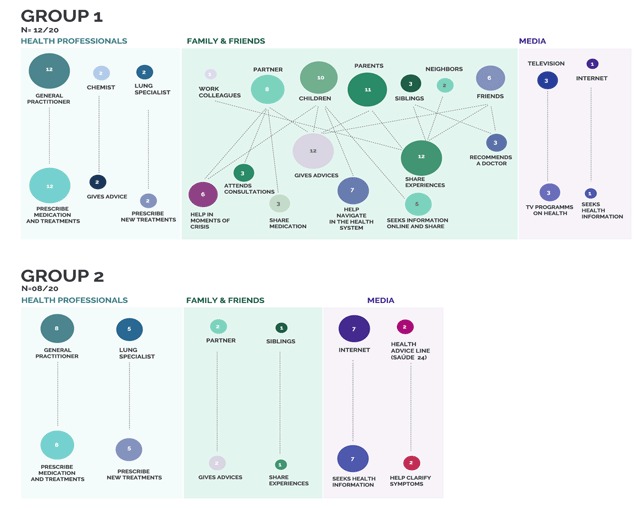
Map of health literacy mediators and practices according to awareness narratives. ^1^ Circles are the frequency that each mediator was mentioned and lines are the association to different health literacy practices.

This group displayed a dense network of health literacy mediators, constituted mainly by relatives and the primary care physician (Figure 1). Close family members emerged as health mediators with whom they have strong ties, providing emotional support (both moral support and caring) and pragmatic support (access to information and knowledge) through sharing of experiences, advice, help navigating health systems, and sharing medication [1.3.1a; 1.3.1b]. Partners and children also share the task of managing the patient’s condition, specifically watching out for symptoms, reminding them to take the medication, and helping in moments of crisis [1.3.1c; 1.3.1d]. Some interviewees also mentioned emotional support provided by friends and neighbors trying to help in moments of crisis [1.3.1e] or inquiring about treatments and consultations [1.3.1f].

The primary care physician was mentioned by all the interviewees as a source of instrumental support, providing tangible aid and services. The relationships with these health professionals relied on frequent interactions [1.3.2a], in which most patients were followed by the same primary care physician for a long time. Interviewees valued the quality of clinical encounters, which result in consistency of information [1.3.2b], personalized conversations, and trust and satisfaction with healthcare services – with the establishment of a “genuine” concern and “close relationship” [1.3.2c].

Few interviewees referred to the media (Internet and television) as health mediators, mentioning difficulties in distinguishing trustworthy and reliable information [1.3.3a; 1.3.3b].

### Narrative of disruption

#### Dealing with an asthma diagnosis

Participants mentioned the highly disruptive impact of asthma in everyday life, highlighting changes in the way they “see life” [2.1a]. They mentioned “upsetting” limitations when practicing sport or participating in other leisure activities [2.1b; 2.1c] and feelings of stigma associated with others’ negative reactions upon witnessing episodes of crises [2.1d].

Interviewees reported several attempts to preserve a public image in which asthma is kept hidden and symptoms are less obvious. Elsa, for example, recognized that her son (also asthmatic) wanted to hide asthma from his colleagues, and never took medication to school [2.1e]. In some cases, interviewees have trouble understanding why asthma appeared for them but not to their friends or partners who engaged in risk behaviors for asthma (e.g. smoking) [2.1f].

#### Self-management skills

Interviewees revealed a reactive approach to asthma management, feeling the need to address their asthma only when symptoms increased or significantly impacted their daily life. Taking preventive medication was not maintained, alternative and complementary solutions and coping strategies were used to control anxiety, as for instance, through praying [2.2a] or acupuncture [2.2b]. Reading the patient information leaflet was one of the main reasons why participants showed reluctance adhering to medication [2.2c].

Some interviewees expressed feelings of personal guilt stating they did not make enough income to improve their asthma status [2.2d]. Lack of knowing how to recognize the triggers of asthma also led to limiting their life in ‘asthma periods’, such as avoiding going to the beach [2.2e]. Experiencing asthma crises and not being able to control it led to more visits to the emergency rooms [2.2f], as well as to an excessive intake of quick relief medication [2.2g].

#### Health literacy mediators

This group of interviewees had a restricted network of core mediators (Figure 1). It included experts working on the healthcare system (the primary care physician, lung specialists), with whom they had strong ties, and the media (internet and health advice hotline *Saúde 24*), making up weak ties, providing informational support such as access to specialized health information [2.3.1a]. Nonetheless, the communication with health professionals was not always described as effective, which limited patient engagement and a more active participation on treatment [2.3.1b; 2.3.1c]. Commonly, when people felt isolated symptoms – which in their opinion did not justify a visit to the doctor – they tended to search online for a solution, but were usually skeptical about the information available [2.3.2a].

Family members who were emotionally close rarely emerged as health mediators and they were not always successful in providing support [2.3.3a]. That gave rise to patients feeling that they lacked support to help manage the condition while, in the narratives of some interviewees, they assumed the responsibility of helping the others [2.3.3b].

## Discussion

This study reveals several features that can be useful in integrated people-centred health services for asthma. It helps in clarify responsibility-shifting between main health mediators, enabling effective collaboration between health professionals (in particular the primary care physician and lung specialists), family and friends, and media (especially the Internet), through the identification of their roles and level of centrality in supporting asthma management in relation to two main awareness narratives – minimization versus disruption. These narratives are characterized by distinct reactions to diagnosis – low impact versus disruption/feelings of stigma, different approaches to self-management skills – control versus reactive/alternative solutions, and dependence on diverse types of support – instrumental by primary care physician /pragmatic and emotional by relatives within dense networks versus informational by primary care physician and Internet within restricted networks. The ties established with each mediator are stronger when interactions are more frequent (intensity of ties), when it is easier to make contacts (dispersion), and when the information is comprehensible, consistent and reliable. This finding draws attention to the importance of exploring interactional factors, in particular patient- primary care physician relationships, to understand patient’s distributed health literacy. Considering that patients showed they were not confident in relying and trusting in information found online, this feature also supports the need to assess the quality of asthma-related websites, in a context where improving the quality of health-related websites have the potential to improve health literacy of general population [39]. Finally, this study draws attention to the challenge of care for people with chronic complex needs and how care should be addressed at the patient in his/her social network and local community, aiming at understanding how sharing responsibilities works and how concepts such as ‘engaging the community’ and ‘distributed health literacy’ explain compliance of patients in care.

A narrative of minimization tended to be activated by those who ‘accept’ the identity of ‘asthmatic’, which contrasts with the disruptive narrative of ‘deniers’. These identities have been previously identified by Adams [40] among patients with asthma, not disclosing any particular link between attitudes towards asthma and SES. Narratives correspond to acceptance or rejection of their condition and are associated with different attitudes towards medication, disease management and coping strategies. What this study adds to the literature is the idea that these identities are connected to different configurations of social networks of each individual, which highlights the importance of analyzing patients’ narratives to trace the tapestry that compose them. Core network of health mediators provided most of health literacy competencies (e.g. by giving advice, preventing exposure to certain environments or preventing symptoms, helping in moments of crisis and intake of quick relief medication, sharing experiences, helping with coping strategies, understanding and obtaining information about asthma, seeking online information, or recommending a doctor). Thus, planning feasible asthma self-management regimens needs us to approach health literacy as a distributed attribute and not exclusively individual [41,42]. Improving interactions between health mediators while clarifying the distribution of responsibilities among those involved in shared health literacy practices would create health benefits [43] that may also extend to the social domain [44].

The fact that participants did not make reference to structural support provided by peers who were non-relatives, such as friends with asthma and family members without asthma – who have reported difficulties in advising asthmatics – points to the need of promoting education groups with peers and family members, especially for those without asthma in their close family. That is typically the case of those with a disruptive narrative. In fact, transgenerational paths of chronic illness minimize the negative impacts that psychosocial difficulties might have on self-management strategies [45,46]. Additionally, through the analysis of the coping strategies and resilience patterns of each patient, a collective understanding of illness susceptibility and its effects in lessening the disruptive potential towards one’s biography might be achieved [47,48]. It is possible that by relying on these models people may employ adequate self-management and effectively integrate of asthma into their lives and identity [49,50]. These prototypes may also help turn disruptive assertions towards normalcy, in the form of “restitution narratives”, which prevent the threat of identity discontinuity imposed by chronic illness [51]. What makes this proposal particularly relevant is the existence of the narrative of disruption in asthmatics diagnosed in childhood. That contradicts the assumption that diagnosis in childhood would raise confidence over time. Those diagnosed since childhood instead, show some ‘saturation’ regarding asthma and lower adherence to medication and treatments [52]. This observation underlines the importance of patient counseling and peers education groups, specifically on the transition periods (from childhood to adolescence and adulthood) when compliance tends to fail more due to patient-related determinants [53,54].

This study is not without limitations and some issues that became apparent were the gendered narratives of the experience of care of self-management, as well as the limitation of our sample size, and the cross-sectional nature of the study design. Recall bias may also had influenced interviewees’ narratives, as patients recalled experiences extending as far back as to diagnosis, for some interviewees, since childhood.

In conclusion, this study stresses the need for addressing distributed health literacy through narratives that show patients’ awareness, in order to identify the diversity of roles performed by core health mediators in support for asthma management. Findings helped to develop recommendations for integrated asthma care (**Box 2**) in particular by clarifying the boundaries of responsibility-shifting between health mediators and patients though two main viewpoints: firstly, identifying networks and types of support on asthma daily management; and secondly, exploring the interactional factors to understand patient attitudes towards asthma. However, future research is needed to assess how networks configurations change over time and how types of support are affected through life-course.

**Box 2: Recommendations for an USF with Integrated Asthma Care Provision**

1. Annual reassessment of the health status of the patient, focusing on the frequency of asthma symptoms and update medical prescriptions (considering the dosage, duration and adverse effects of the treatment and medication).
2. To explore reactions to diagnosis (acceptance or rejection) and to identify health mediators in an asthmatic’s tasks.
3. To clarify responsibility-shifting between main health mediators, enabling effective collaboration between them through the creation of hybrid spaces for dialogue.
4. Personalized educational interventions to help acceptance and minimize negative impacts that psychosocial difficulties might have on self-management strategies.
5. To enrich support provided by peers’ education groups, especially on the transition periods (from childhood to adolescence and adulthood) and for those without asthma in their close family.
6. To assess the quality of asthma-related websites.
